# Dataset on the epidemiology and genetic diversification of dengue virus (DENV) serotypes and genotypes in Mexico

**DOI:** 10.1016/j.dib.2020.106077

**Published:** 2020-07-29

**Authors:** Eduardo Domínguez-de-la-Cruz, María de Lourdes Muñoz, Ericel Hernández-García, Gerardo Pérez-Ramírez, Randy E. David, Joel Navarrete-Espinosa, Álvaro Díaz-Badillo, Miguel Moreno-Galeana, Cesar Armando Brito-Carreón

**Affiliations:** a Department of Genetics and Molecular Biology, Center for Research and Advanced Studies of the National Polytechnic Institute (CINVESTAV—IPN), Mexico City, Mexico; b Laboratories of Biological Anthropology, University of Kansas, Lawrence, KS, USA; c Epidemiology Division, Coordination of Integrated Health Programs, Mexican Social Security Institute, Mexico City, Mexico; d University of Texas Rio Grande Valley, Department of Human Genetics and South Texas Diabetes and Obesity Institute, TX, USA

**Keywords:** Dengue fever, DENV genotypes, DENV serotypes, Epidemiology, Evolutionary virology, Introduction of novel genotypes, Phylogeny, Severe dengue

## Abstract

Dengue virus (DENV) evolution has had a significant impact on disease pathogenesis, virulence, and epidemiology in Mexico. Novel genotypic variation in DENV serotypes and genotypes may influence the magnitude and severity of dengue epidemics, as evidenced by 2009 data from Veracruz State. The data presented herein is related to the publication entitled “Epidemiological Implications of the Genetic Diversification of Dengue Virus (DENV) Serotypes and Genotypes in Mexico” [1]. Raw data and trees provide epidemiological data on DENV prevalence and a comprehensive phylogeny of both representative sequences collected from an NCBI repository, and 28 additional isolates from acute-phase plasma samples diagnosed with dengue fever or severe dengue (Raw sequencing data is hosted in the public repository Mendeley Data (http://dx.doi.org/10.17632/bf2kdhhf6x.2). Phylogenetic trees for each DENV serotype (DENV-1, -2, -3 and -4) were constructed using these sequences by a maximum likelihood methodology as well as a Bayesian Markov chain Monte Carlo (MCMC) integration approach. Phylogenetic trees exhibited: (1) DENV-1, genotype V, (2) the DENV-2 Asian/American and Asian II genotypes, (3) DENV-3, genotype III, and (4) DENV-4, genotype I. This data can be beneficial for future analyses on DENV serotype and genotype structure and the introduction of novel DENV genotype sequences in the Americas, for the further elucidation of dengue etiology.

**Specifications table**

**Table:** 

**Subject**	Virology
**Specific subject area**	Dengue virus and the introduction of novel DENV genotypes in Veracruz State, Mexico.
**Type of data**	Data are in raw format and have been analyzed. An Excel spreadsheet and figures have been uploaded. Raw sequencing data hosted in the public repository Mendeley Data (http://dx.doi.org/10.17632/bf2kdhhf6x.2) [2].
**How data was acquired**	Raw sequencing data is hosted in the public repository Mendeley Data (http://dx.doi.org/10.17632/bf2kdhhf6x.2) [2]. This data was used to construct phylogenetic trees using divergence time (tMRCA) and rate of nucleotide substitution, built using a Bayesian Markov chain Monte Carlo (MCMC) integration approach, as implemented in BEAST 1.10.3 software [3]. A database of DENV representative sequences were collected freely from the NCBI repository: https://www.ncbi.nlm.nih.gov/genbank/ Raw DENV isolate data compiled freely from the GenBank database from both Veracruz State, and Mexico, as well as other countries, in addition to Mexican DENV epidemiological data from the Mexican SINAVE (Sistema Nacional de Vigilancia Epidemiologica de la Dirección General de Epidemiología/Epidemiological Surveillance Single Information System) (Table 1) were acquired [4]. One Excel spreadsheet with pertinent raw data has been uploaded freely (Supplementary Table 1).
**Data format**	Raw sequencing data is hosted in the public repository Mendeley Data (http://dx.doi.org/10.17632/bf2kdhhf6x.2) [2]. Data are in raw format and have been analyzed. Two tables have been uploaded, Table 1 and Supplementary Table 1 (Excel spreadsheet).
**Parameters for data collection**	RNA obtained from the isolates of DENV-1, -2, -3, and -4, were amplified and sequenced [1]. Raw sequencing data hosted in the public repository Mendeley Data (http://dx.doi.org/10.17632/bf2kdhhf6x.2) [2] were used to construct phylogenetic trees for all DENV serotypes using the maximum likelihood method [5] and Bayesian MCMC approach [6]. Dengue incidence was calculated by the equation: Number of dengue cases in one epidemiological year /100,000 individuals.
**Description of data collection**	Epidemiological data of DENV (Table 1) collected freely from the Mexican SINAVE [4] displays the number of cases, incidence, and percentage of variation in dengue and severe dengue for both Mexico and Veracruz State between 1990 and 2019. The database of DENV sequences hosted in the public repository Mendeley Data (http://dx.doi.org/10.17632/bf2kdhhf6x.2) [2] includes 513 sequences collected from an NCBI repository and 28 sequences obtained from the isolates from Veracruz, Mexico. Database details (Supplementary Table 1) includes: GenBank accession number, URL: GenBank, strain or isolate, year collected, serotype, genotype, country, ID in phylogenetic analyses, and reference(s).
**Data source location**	Center for Research and Advanced Studies of the National Polytechnic Institute (CINVESTAV—IPN), Mexico City, Mexico. Latitude and longitude for collected sample/data 19° 30′ 33″N, 99° 07′ 46″W
**Data accessibility**	Access options located with article. Raw sequencing data and alignment information data is hosted in the public repository Mendeley Data (http://dx.doi.org/10.17632/bf2kdhhf6x.2[2]; a description of each DENV sequence is displayed in Supplementary Table 1, and epidemiological raw data is displayed in Table 1.
**Related research article**	Author's names: Hernández-García, María de Lourdes Muñoz, Randy E. David, Gerardo Pérez-Ramírez, Joel Navarrete-Espinosa, Álvaro Díaz-Badillo, Eduardo Domínguez-de-la-Cruz, Miguel Moreno-Galeana, Cesar Armando Brito-Carreón Title: Epidemiological Implications of the Genetic Diversification of Dengue Virus (DENV) Serotypes and Genotypes in Mexico Journal: INFECTION, GENETICS AND EVOLUTION Volume 84, October 2020, 104391. [https://doi.org/10.1016/j.meegid.2020.104391]

**Value of the data**

• This data will serve as a reference for future analyses on the incidence, epidemiology, and introduction of novel DENV genotypes/serotypes in Mexico—fostering a greater understanding of the emergence of variable dengue strains in the Americas.
• All institutions involved in public health, disaster relief, and arbovirus control programs can benefit from this data through a more nuanced understanding of the association between specific genotypes (within serotypes) and presentation of disease.
• Novel DENV sequence data from Mexico can aid the future phylogenetic classification of dengue serotypes and genotypes circulating in the Americas, useful to understanding virus evolution and the association between genotype/serotype and disease pathogenesis.
• DNA sequencing and serotype identification of DENV samples in Veracruz State and elsewhere in Mexico can provide crucial supporting data for future control programs, re-emergence research, source/sink studies, transnational transmission pattern analysis, and herd immunity research.
• The epidemiological analysis of dengue virus in Veracruz State in 2009 can provide a foundation for further insight into the interrelationship between climatological factors, viral evolution, and disease distribution.

## 1 Data description

**Fig. 1:**
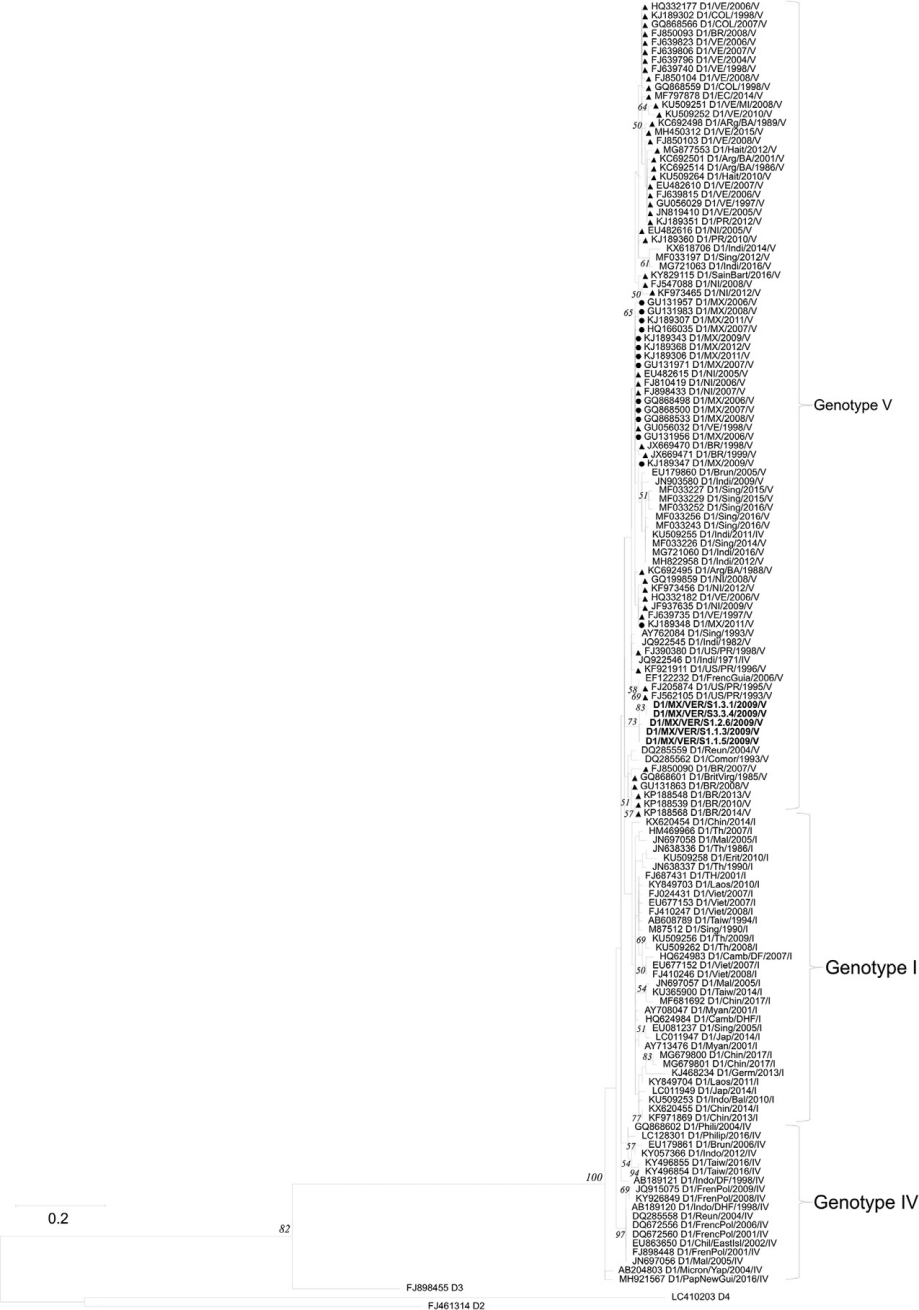
Maximum-likelihood phylogenetic tree of 146 DENV-1 *NS3* (141 bp) gene sequences constructed using raw data hosted in the public repository Mendeley Data (http://dx.doi.org/10.17632/bf2kdhhf6x.2) [2]. The percentage of replicate trees in which the associated taxa clustered together in the bootstrap test (1,000 replicates) are shown next to branches. This phylogenetic tree includes five novel sequences clustered within genotype V, labelled in bold. Positions of sequences from Mexico are indicated using a dot, and those from the Americas using a triangle. Horizontal branch lengths are proportional to the bar representing number of nucleotide substitutions/sites. DENV strains are named as follows: GenBank accession number/strain/country/year/serotype/genotype.

**Fig. 2:**
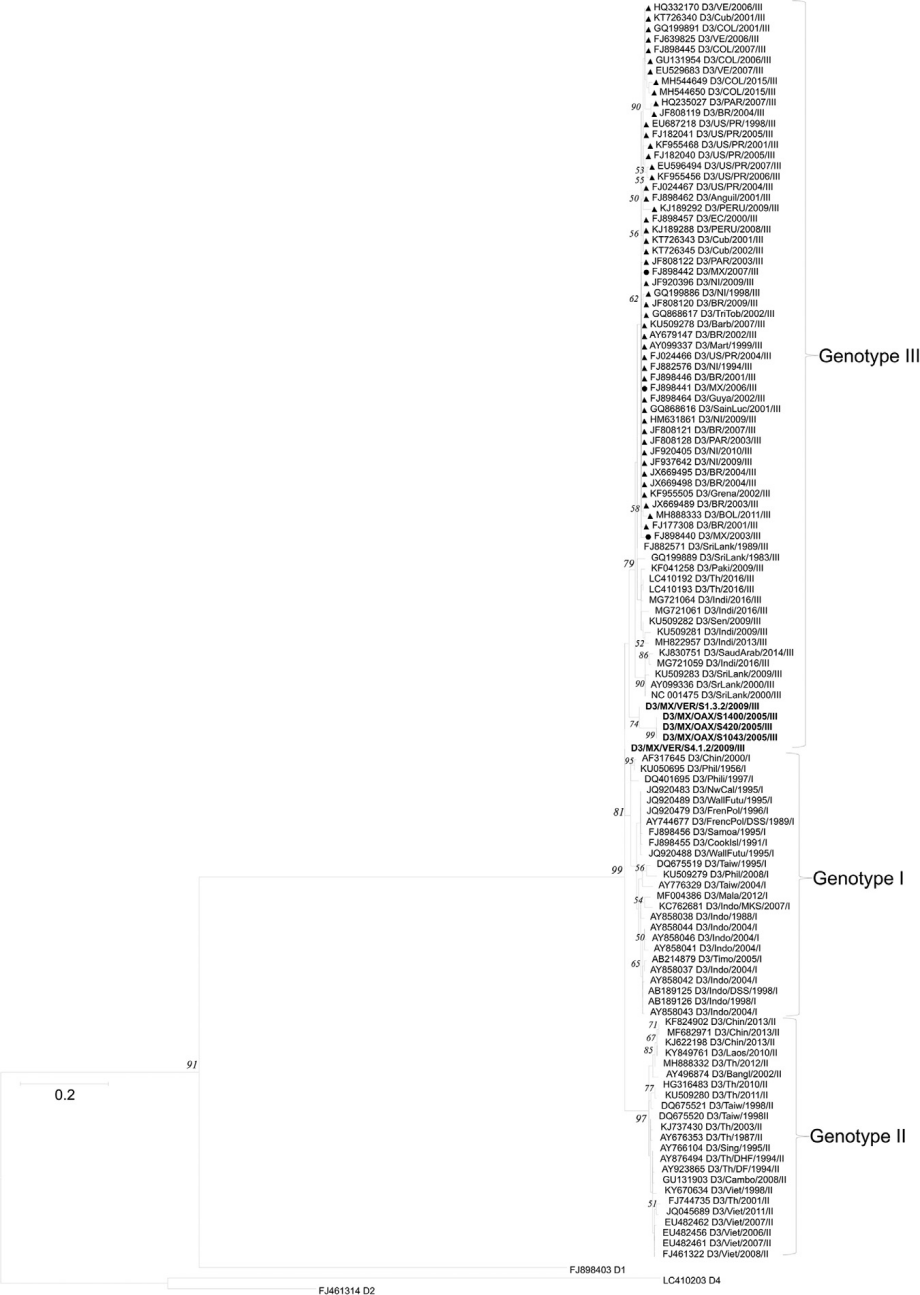
Maximum-likelihood phylogenetic tree of 119 DENV-3 *NS3* (249 bp) gene sequences constructed using raw data hosted in the public repository Mendeley Data (http://dx.doi.org/10.17632/bf2kdhhf6x.2) [2]. The percentage of replicate trees in which the associated taxa clustered together in the bootstrap test (1,000 replicates) are shown next to branches. This phylogenetic tree includes two novel sequences clustered within genotype I, labelled in bold. Positions of sequences from Mexico are indicated using a dot, and those from the Americas using a triangle. Horizontal branch lengths are proportional to the bar representing number of nucleotide substitutions/sites. DENV strains are named as follows: GenBank accession number/strain/country/year/serotype/genotype.

**Fig. 3:**
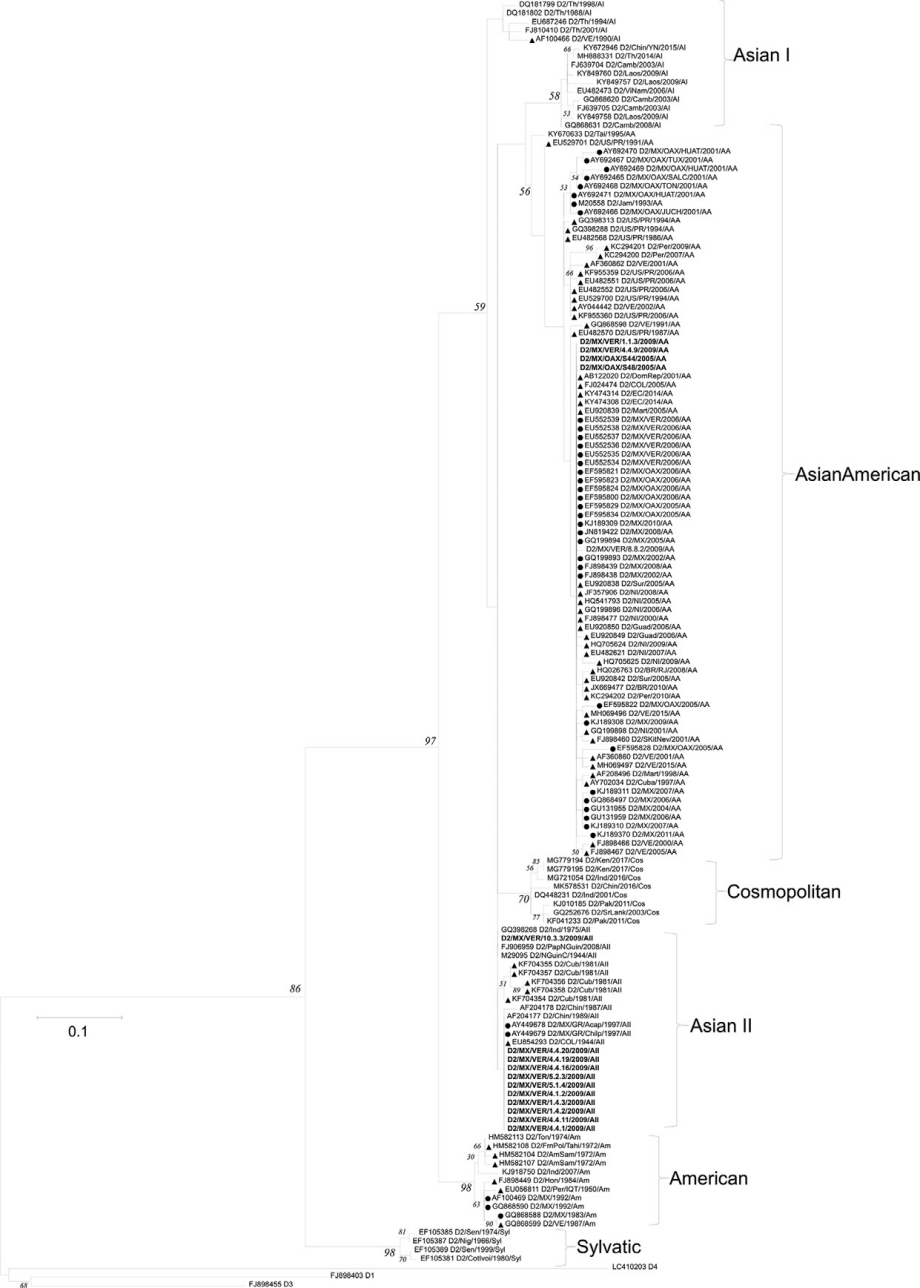
Maximum-likelihood phylogenetic tree of 146 DENV-2 C-*prM* (134 bp) sequences constructed using raw data hosted in the public repository Mendeley Data (http://dx.doi.org/10.17632/bf2kdhhf6x.2) [2]. The percentage of replicate trees in which the associated taxa clustered together in the bootstrap test (1,000 replicates) are shown next to branches. This phylogenetic tree includes four novel sequences clustered within the Asian/American genotype, and ten novel sequences clustered within the Asian II genotype (labelled in bold). Positions of sequences from Mexico are indicated using a dot, and those from the Americas using a triangle. Horizontal branch lengths are proportional to the bar representing number of nucleotide substitutions/sites. DENV strains are named as follows: GenBank accession number/strain/country/year/serotype/genotype. MX: Mexico; MZ/OAX: Mexico, Oaxaca; MX/VER: Mexico, Veracruz; US/PR: United States, Puerto Rico; VE: Venezuela; NI: Nicaragua; COL: Colombia; EC: Ecuador; BR: Brazil.

**Fig. 4:**
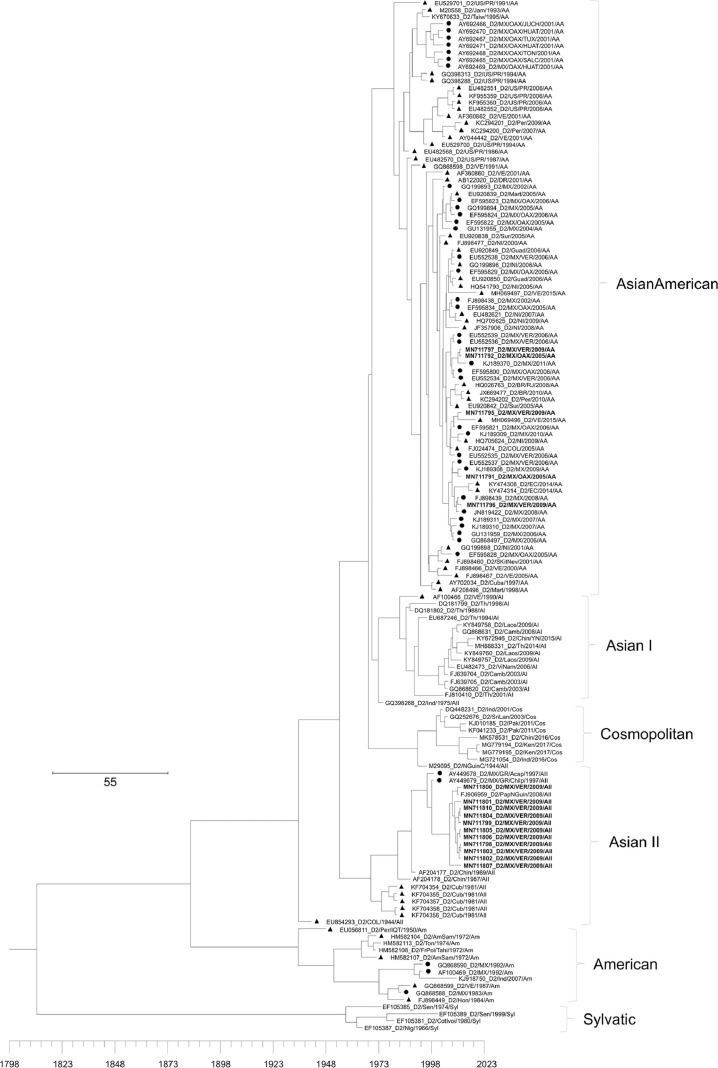
Maximum clade credibility (MCC) of 146 DENV-2 C-*prM* (134 bp) sequences constructed using raw data hosted in the public repository Mendeley Data (http://dx.doi.org/10.17632/bf2kdhhf6x.2) [2]. This phylogenetic tree includes five novel sequences clustered within the Asian/American genotype, and eleven novel sequences clustered within the Asian II genotype (labelled in bold). Positions of sequences from Mexico are indicated using a dot, and those from the Americas using a triangle. Horizontal branch lengths are proportional to the bar representing the probability of coalescence. DENV strains are named as follows: GenBank accession number/serotype/country/year/genotype.

**Fig. 5:**
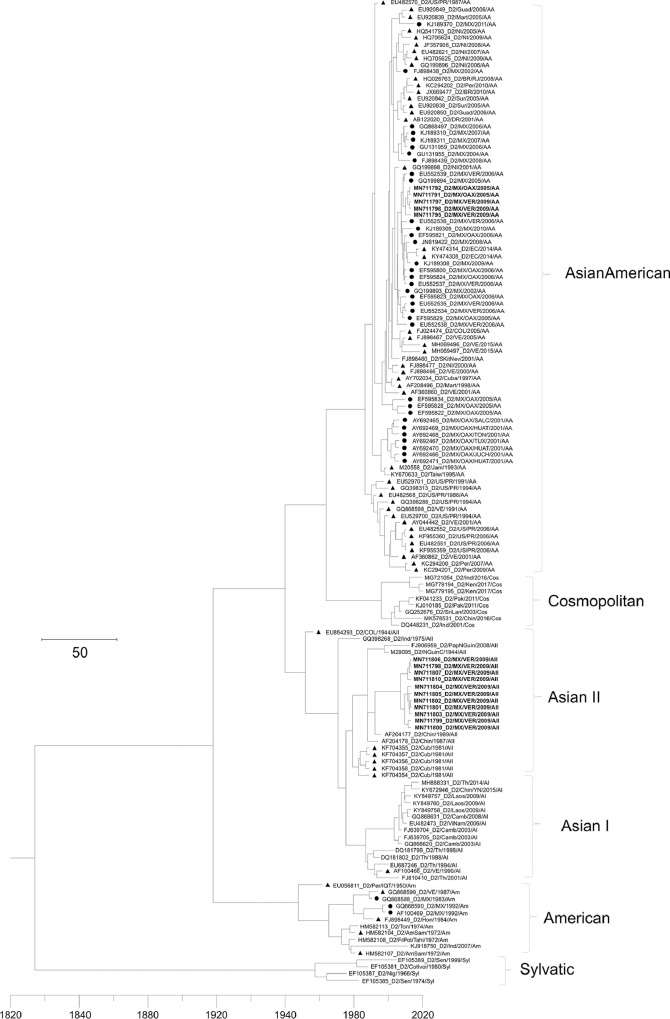
Maximum clade credibility (MCC) tree of 144 DENV-2 C-*prM* (384 bp) gene sequences constructed using raw data hosted in the public repository Mendeley Data (http://dx.doi.org/10.17632/bf2kdhhf6x.2) [2]. Details of phylogenetic tree analysis and graphical features are the same as those provided in the Fig. 4 caption. This phylogenetic tree includes five novel sequences clustered within the Asian/American genotype, and eleven novel sequences clustered within the Asian II genotype (labelled in bold). Positions of sequences from Mexico are indicated using a dot, and those from the Americas using a triangle. MX: Mexico; MZ/OAX: Mexico, Oaxaca; MX/VER: Mexico, Veracruz; US/PR: United States, Puerto Rico; VE: Venezuela; NI: Nicaragua; COL: Colombia; EC: Ecuador; BR: Brazil.

**Fig. 6:**
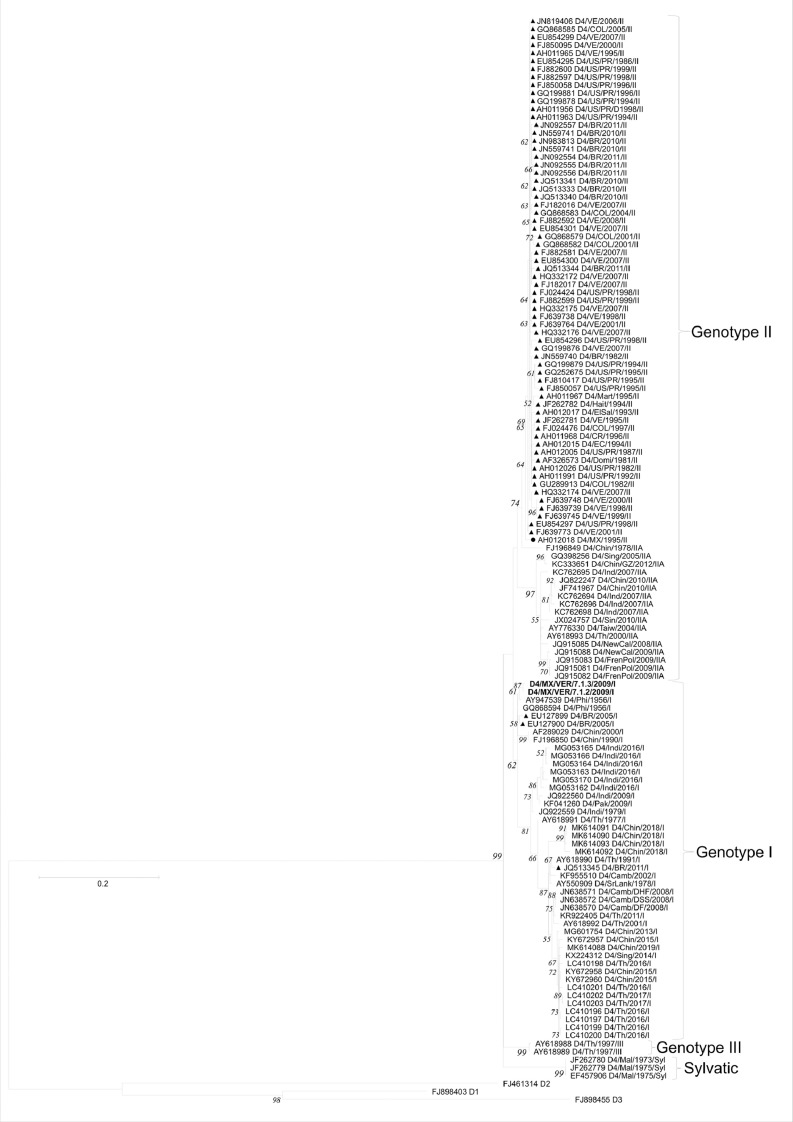
Maximum-likelihood phylogenetic tree of 132 DENV-4 *C-prM* (363 bp) gene sequences constructed using raw data hosted in the public repository Mendeley Data (http://dx.doi.org/10.17632/bf2kdhhf6x.2) [2]. The percentage of replicate trees in which the associated taxa clustered together in the bootstrap test (1,000 replicates) are shown next to branches. This phylogenetic tree includes two novel sequences clustered within genotype I, labelled in bold. Positions of sequences from Mexico are indicated using a dot, and those from the Americas using a triangle. Horizontal branch lengths are proportional to the bar representing number of nucleotide substitutions/sites. DENV strains are named as follows: GenBank accession number/strain/country/year/serotype/genotype.

**Fig. 7:**
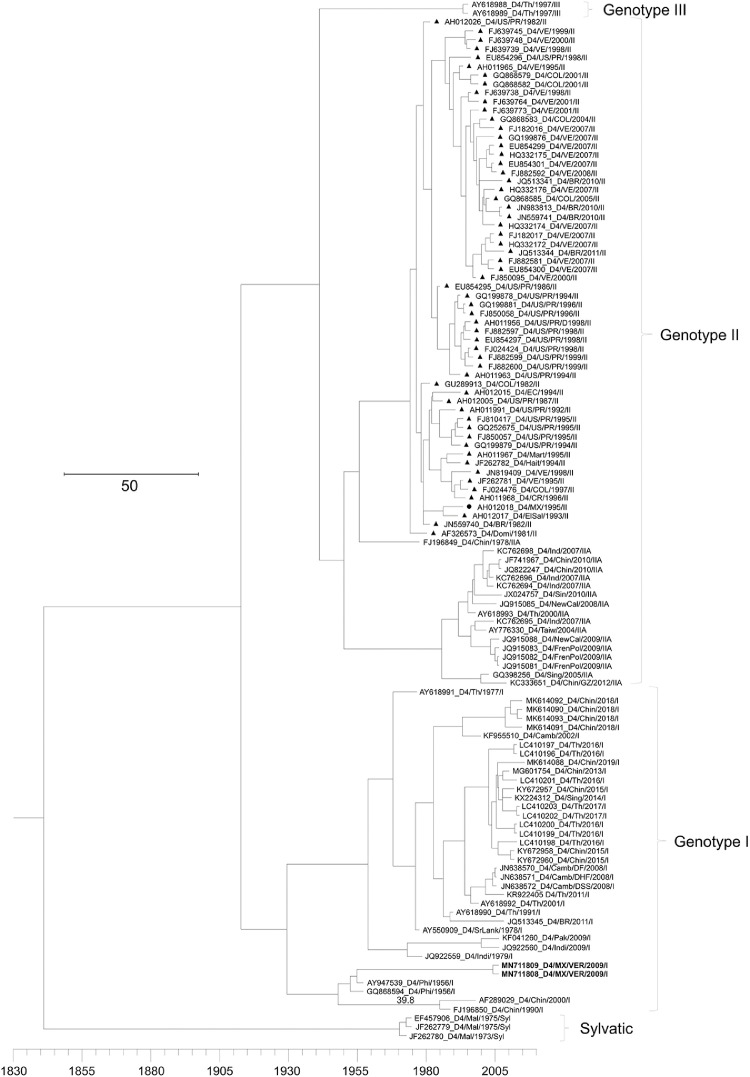
Maximum clade credibility (MCC) phylogenetic tree of 118 DENV-4 *C-prM* (567 bp gene sequences) constructed using raw data hosted in the public repository Mendeley Data (http://dx.doi.org/10.17632/bf2kdhhf6x.2) [2]. This phylogenetic tree includes two novel sequences clustered within genotype I, labelled in bold. Positions of sequences from Mexico are indicated using a dot, and those from the Americas using a triangle. Horizontal branch lengths are proportional to the bar representing the probability of coalescence. DENV strains are named as follows: GenBank accession number/serotype/country/year/genotype.

Table 1 displays epidemiological data of DENV in both Mexico and Veracruz State specifically, between 1990 and 2019. Characteristics of the DENV sequences used to construct phylogenetic trees from Mexico and Veracruz State (GenBank accession number: EF589666 – EF589668 and MN711791 – MN711815) and additional representative sequences from the NCBI repository are presented in Supplementary Table 1, which also includes: GenBank accession number, URL: GenBank, strain or isolate, year collected, serotype, genotype, country, and ID in phylogenetic analyses. Alignment and sequences for DENV-1, -2, -3, and -4 are hosted in the public repository Mendeley Data (http://dx.doi.org/10.17632/bf2kdhhf6x.2) [2]. Database details (Supplementary Table 1) includes: GenBank accession number, URL: GenBank, strain or isolate, year collected, serotype, genotype, country, ID in phylogenetic analyses, and references associated with each sequence. The phylogenetic trees for DENV-1, -2, -3, and -4 were constructed using the sequences of either the *C-prM* or *NS3* gene regions. Fig. 1 displays the phylogeny of *NS3* gene sequences for DENV-1. Sequences from Veracruz State were grouped within genotype V (labelled in bold), similar to other isolates from Latin America. The phylogeny of *NS3* gene sequences for DENV-3 from both Veracruz and Oaxaca States (2009 and 2005 epidemics, respectively) presented as genotype III (labelled in bold; Fig. 2). The phylogenetic tree for DENV-2 illustrates isolates of the DENV-2 Asian/American genotype related to strains from Oaxaca (Fig. 3). The DENV-2, Asian II genotype was shown to share a genetic affinity with dengue strains from Chilpancingo, and Acapulco, Mexico, as well as Colombia, Cuba, and China, both according to a maximum composite likelihood (MCL) methodology (Fig. 3) and a Bayesian Markov chain Monte Carlo (MCMC) approach (Fig. 4) [3]. The Bayesian trend moreover held true for Fig. 5, where 384 instead of 134 nucleotides were used, omitting sequences of Guerrero State samples due to their relatively shorter length. DENV-4 isolates clustered with genotype I, sharing a recent common ancestor with strains from the Philippines and Brazil (Fig. 6). Lastly, DENV-4 isolates, according to a Bayesian model, were found to cluster with isolates from China after omitting Brazilian sequences due to their inherently shorter length (Fig. 7).

**Table 1:** Raw data of the epidemiology of DENV in Mexico and Veracruz State. Number of cases and percentage of variation of dengue and severe dengue in both Mexico and Veracruz State between 1990 and 2019 are displayed.

Number of cases					Percentage of variation*			
YEAR	D-MEX	SD-MEX	D-VER	SD-VER	D-MEX	SD-MEX	D-VER	SD-VER
**1990**	1663	6	160	0	0%	0%	0%	0%
**1991**	1931	1	143	0	16%	-83%	-11%	0%
**1992**	1102	0	32	0	-43%	-100%	-78%	0%
**1993**	791	0	166	0	-28%	0%	419%	0%
**1994**	7868	0	2462	0	895%	0%	1383%	0%
**1995**	14396	355	5503	79	83%	0%	124%	0%
**1996**	19835	884	5297	358	38%	149%	-4%	353%
**1997**	51021	954	10563	155	157%	8%	99%	-57%
**1998**	15181	225	2147	28	-70%	-76%	-80%	-82%
**1999**	23725	220	2331	10	56%	-2%	9%	-64%
**2000**	1706	50	568	7	-93%	-77%	-76%	-30%
**2001**	4643	312	2344	14	172%	524%	313%	100%
**2002**	13131	2159	2357	98	183%	592%	1%	600%
**2003**	5018	1419	988	95	-62%	-34%	-58%	-3%
**2004**	6243	1959	4250	1570	24%	38%	330%	1553%
**2005**	17487	4418	3901	636	180%	126%	-8%	-59%
**2006**	22566	4426	7265	1066	29%	0%	86%	68%
**2007**	42936	9433	12608	2645	90%	113%	74%	148%
**2008**	27964	7560	2066	2051	-35%	-20%	-84%	-22%
**2009**	44565	11396	3412	2978	59%	51%	65%	45%
**2010**	22352	9336	867	302	-50%	-18%	-75%	-90%
**2011**	10970	4608	996	651	-51%	-51%	15%	116%
**2012**	32662	17706	7531	5041	198%	284%	656%	674%
**2013**	43663	18667	4941	3858	34%	5%	-34%	-23%
**2014**	23374	8647	2060	1866	-46%	-54%	-58%	-52%
**2015**	21201	5464	2884	876	-9%	-37%	40%	-53%
**2016**	14112	3683	1833	391	-33%	-33%	-36%	-55%
**2017**	11334	2794	1031	324	-20%	-24%	-44%	-17%
**2018**	8229	4477	2239	467	-27%	60%	117%	44%
**2019**	27884	13621	9195	1707	239%	204%	311%	266%

D-MEX: dengue in Mexico; SD-MEX: severe dengue in Mexico; D-VER: Dengue in Veracruz; SD-VER: severe dengue in Veracruz; *: indicates the percentage of variation in the present year compared with the previous year.

## 2 Experimental design, materials and methods

### 2.1 Epidemiology of dengue in Mexico

The data source of DEN incidence and number of cases in Mexico between 1990 and 2019 was the Mexican SINAVE [4].

### 2.2 Sample collection

Sixty-five isolates of DENV viruses were obtained from acute-phase plasma collected from patients with dengue fever or severe dengue through the “Instituto Mexicano del Seguro Social” (IMSS) from Veracruz State in 2009. Samples were anonymous, with only information regarding disease symptomology provided. Excess samples that remained after diagnostic testing were stored at −70 °C. The project was reviewed and approved by the Institutional Review Board of the IMSS (Commission of Scientific Research) and the Bioethical Commission for Research in Humans of Center for Research and Advanced Studies of the National Polytechnic Institute (Comité de Bioética Para la Investigación en Seres Humanos, COBISH-CINVESTAV).

### 2.3 Dengue virus isolation

*Aedes albopictus* clone C6/36 cells were grown in minimal essential media (MEM), supplemented with 10% fetal bovine serum (FBS) and nonessential amino acids. Cells were maintained at 28 °C without carbon dioxide (CO2). After 18 h of culture, cells (2 × 10^6^/100 mm plate) were infected with 0.2 ml DENV-2 inoculum with an input MOI of 600 PFU/cell and were incubated at 28°C for 10 days.

Viruses were isolated as previously described [7] with a few modifications. After 18 hours of culture, C6/36 cells (2 × 10^6^/15 ml tube) were infected with 0.01 to 0.1 ml of serum specimen per tube, diluted to 1.0 ml with medium, and incubated for 2 h at 28 °C. After one wash, 3.0 ml of MEM was added, and cells were cultivated for approximately 15 days at 28 °C (passage number 1). Cells were observed daily and when a cytopathic effect was apparent from syncytium formation and cellular lysis, the cells were harvested and centrifuged at 3,000 rpm for 5 min. The pellet was then suspended in 0.6 ml of MEM and stored in aliquots of 0.15 ml at –70 °C. The supernatant (approximately 2.5 ml) was stored in 2 aliquots of 1.0 ml and one aliquot of 0.5 ml, at –70 °C. To obtain passage numbers two and three, C6/36 cells were incubated with 1.0 ml of the supernatant obtained from earlier passages, for 2 h at 28 °C following the same procedure as above. Serotypes in all samples were determined based on the isolates obtained from the first, second, or third culture passages.

### 2.4 RNA extraction

RNA was extracted from the cell culture supernatant using TRIzol™ LS reagent, Cat. No. 10296028 (Gibco, Gaithersburg, MD) according to the manufacturer's recommendations. Isopropanol-precipitated RNA was recovered by centrifugation, then air-dried. The resultant RNA pellet was suspended in 50 μl of diethyl pyrocarbonate (DEPC) treated water, Cat. No. 46–2224 (Invitrogen Life Technologies, Carlsbad, CA) and used as a template for reverse transcription with polymerase chain reaction (RT-PCR).

### 2.5 Reverse transcription-polymerase chain reaction (RT-PCR)

The RT-PCR protocol previously described [8] was used to discern DENV serotypes. The following genes were then amplified and sequenced: protein C—nucleotide 139 (C-139) to prM-789 (prM-789) [4]; and NS3 from nucleotides 4,899 (DV1) to 5,067 (DSP1), 4,899 (DV1) to 5,279 (DSP2), 4,899 (DV1) to 5,174 (DSP3) or 4,899 (DV1) to 5,342 (DSP4), for serotypes DENV-1, DENV-2, DENV-3 and DENV-4, respectively [8]. All assays were performed using the SuperScript™ III One-Step RT-PCR System with Platinum™ Taq DNA Polymerase, Cat. No. 12574026 (Invitrogen/Thermo Fisher Scientific, Waltham, Massachusetts). A mixture of 5 μl of total RNA (0.1–0.5 μg), and 1 pmol/µl of each primer at nucleotide positions C-139 (forward, 5′-CAATATGCTGAAACGCGHG-3′) and prM-789 (reverse, 5′-CCTTCNGMNGACATCC-3′) was incubated at 65 °C for 5 min. After adding 25 μl of 2X Reaction Mix, and 2 μl of SuperScript™ III RT/Platinum™ Taq with DEPC-treated water (total volume: 50 μl), RT was carried out at 50 °C for 60 min. This step was followed by incubation at 94 °C for 2 min to inactivate the reverse transcriptase. Afterwards, Platinum™ Taq was activated by incubation at 94 °C for 2 min, to amplify the 629 bp fragment of the C-pM gene. This was followed by 35 cycles with the following conditions: 94 °C for 30 s, 55 °C for 45 s, 72 °C for 85 s, and a final extension of 72 °C for 10 min (storage at 4 °C).

Serotypification was carried out following the protocol of Seah et al. [8]: 10 cycles of 95 °C for 30 s, annealing at 55 °C for 1 min, and extension at 72 °C for 1 min and 35 cycles of 95 °C for 30 s, 55 °C for 30 s, and 72 °C for 30 s, with a final extension of 72 °C for 7 min (storage at 4 °C). In addition, the NS3 region — nucleotides 4,899 to 5,067 (NS3-169) for DENV-1, 4,899 to 5,279 (NS3-362) for DENV-2, 4,899 to 5,174 (NS3-265) for DENV-3, and 4,899 to 5,342 (NS3-426) for DENV-4 was obtained via RT-PCR assay as aforementioned, but with the following primers:

DV1 (forward, 5′-GGRACKTCAGGWTCTCC-3′) and DSP1 (reverse, 5′-AGTTTCTTTTCCT-AAACACCTCG-3′) for DENV-1; DV1 and DSP2 (reverse, 5′-CCGGTGTGCTCRGCYCTGAT-3′) for DENV-2; and DV1 and DSP3 (reverse, 5′-TTAGAGTYCTTAAGCGTCTCTTG-3′) for DENV-3; and DV1 and DSP4 (reverse, 5′-CCTGGTTGATGACAAAAGTGTTG-3′) for DENV-4.

### 2.6 Sequencing of PCR products

For automated sequencing, spin column-purified QIAquick PCR Purification Kit, Cat. No. 28106 (Qiagen, Chatsworth, CA.) DNA fragments were sequenced using the BigDye™ Terminator v3.1 Cycle Sequencing Kit, Cat. No. 4337458 (Applied Biosystems/ThermoFisher Scientifics, Waltham, MA). Sequencing was conducted using an Applied Biosystems Prism 3100 in a short capillary (47 cm x 50 μm inside diameter), and Performance Optimized Polymer 6, Cat. No. 4352757 (Perkin-Elmer, Waltham, MA, and Applied Biosystems).

### 2.7 Phylogenetic tree construction using raw data

Phylogenetic trees for DENV-1, -2, -3, and -4 were performed using the maximum likelihood method of Tamura and Nei [5]. For this method, the percentage of replicate trees in which the associated taxa clustered together in the bootstrap test (1,000 replicates) are shown next to each branch [9]. Initial trees for the heuristic search were obtained by applying the neighbor-joining method to a matrix of pairwise distances estimated using maximum composite likelihood (MCL). A discrete gamma distribution was used to model evolutionary rate differences among sites with 3 rate categories (+G, parameter = 0.2447). The final tree is drawn to scale, with branch lengths measured by number of substitutions per site. All aforementioned analyses were performed using MEGA7 (Molecular Evolutionary Genetics Analysis Version 7.0) software [10].

The estimation of phylogeny, divergence time (tMRCA) and rate of nucleotide substitution of 2009 DENV-1, -2, -3, and -4 samples from Veracruz State was performed. The different genotypes were assessed according to a Bayesian MCMC approach. The MCMC chain length was run for 30 million generations along with the 95% highest posterior density (HPD) intervals. A GTR + G substitution model (general time-reversible model with four gamma-distributed rate categories) was used for the four sets of different sequences. The uncorrelated log-normal relaxed clock was employed [6] using a constant population size demographic model. The convergence of the chain was evaluated using Tracer 1.7.1 software [11]. Effective sample size (ESS) values of > 200 indicated a sufficient level of sampling. The maximum clade credibility tree was generated using the Tree Annotator program (available in BEAST). Fig Tree 1.4.3 software (http://tree.bio.ed.ac.uk/software/figtree/) was utilized for the visualization of the annotated tree.

## Declaration of Competing Interest

The authors declare that they have no known competing financial interests or personal relationships which have, or could be perceived to have, influenced the work reported in this article.

## Appendix Supplementary materials

Image, application 1

Table S1. Raw data description of dengue virus sequences used in phylogenetic analyses. GenBank accession number, URL: GenBank, strain or isolate, year isolated, serotype, genotype, country, ID in the phylogenetic analysis, and reference(s) are provided in this spreadsheet. Image, application 2
